# Association between trajectories of waist circumference with incidence of type 2 diabetes mellitus, Tehran lipid and glucose study

**DOI:** 10.1038/s41598-026-51739-w

**Published:** 2026-05-13

**Authors:** Maryam Adib, Ladan Mehran, Safdar Masoumi, Fereidoun Azizi, Atieh Amouzegar

**Affiliations:** Endocrine Research Center, Research Institute for Endocrine Disorders, Research Institute for Endocrine Sciences, Shahid Beheshti University of Medical Sciences, No. 23, Parvaneh Street, Velenjak, P.O. Box: 19395-4763, Tehran, Iran

**Keywords:** Waist circumference, Trajectories, Type 2 diabetes mellitus, Obesity, Cohort study, Diseases, Endocrinology, Health care, Medical research, Risk factors

## Abstract

Limited research has addressed the association between the dynamic trends of waist circumference (WC) and the incidence of type 2 diabetes mellitus (T2DM). This study aimed to identify the trajectories of WC and evaluate their association with the incidence of T2DM over an 18-year follow-up among the Iranian population. This study was derived from a large prospective cohort (Tehran Lipid and Glucose Study) and included 6,405 participants aged 20–70 years who were followed up every three years for 18 years; this period was divided into two 9-year intervals to track WC trajectories (measurement period) and record T2DM incidence (outcome assessment period). Three WC trajectory patterns were identified using latent class trajectory analysis. The Cox proportional hazards model was used to evaluate the association between WC trajectories and the risk of developing T2DM. Participants were classified into three WC trajectory patterns of low-increasing (n: 2019), moderate-increasing (n: 3248), and high-increasing (n: 1138). The hazard ratio (HR) of T2DM was 2.3 (95% CI 1.7–2.9) for the high-increasing versus the low-increasing WC trajectory class; the HRs were 2.0 (95% CI 1.1–3.7) in men and 2.5 (95% CI 1.8–3.5) in women. The HR of developing T2DM was 2.6 (95% CI 1.6–4.1) in the normoglycemic in comparison with 1.7 (95% CI 1.0–2.7) in the prediabetic high-increasing WC trajectory class. WC trajectory classes were also compared across baseline body mass index (BMI) categories, which high-increasing WC trajectory class showed the highest risk across all BMI groups. The high-increasing WC trajectory class showed a strong association with the development of T2DM independent of BMI and other important risk factors for T2DM which support continuous surveillance of central obesity for prevention of diabetes.

**Supplementary Information:**

The online version contains supplementary material available at 10.1038/s41598-026-51739-w.

## Introduction

Type 2 Diabetes mellitus (T2DM) represents a growing global public health challenge, with a global prevalence currently at 10.5% and projected to rise to 12.2% by 2045. T2DM prevalence has increased most sharply in middle-income countries, contributing to a substantial economic impact estimated at $966 billion in 2021 and projected to increase to $1,054 billion by 2045^1^. Among the Iranian population, the prevalence of T2DM is similar to the global prevalence of 10.8% ^2^. T2DM is associated with various complications such as cardiovascular disease, retinopathy, neuropathy, nephropathy, lower-extremity amputation, a reduced quality of life, and confers a 1.3 to 2-fold higher mortality risk^3–5^. While genetic predisposition is a key determinant in the development of T2DM, modifiable environmental factors like diet, lifestyle behaviors, physical activity, and air pollution exposure also substantially influence disease risk and present opportunities for effective prevention^6^.

Among various modifiable risk factors, obesity (particularly abdominal obesity) is a significant contributing factor to the development of T2DM^7^. Body mass index (BMI) and waist circumference (WC) are key indicators for assessing general and abdominal obesity, respectively^8,9^. Most evidence indicates that WC has a stronger association with T2DM and is used as a more effective predictor of T2DM onset compared to BMI^10–12^. However, a few studies show different results^13,14^. Consequently, WC emerged as a straightforward metric for assessing abdominal fat in the clinical setting. Also, many studies showed the association of WC and glycemic status and development of T2DM, identifying elevated WC as an independent predictor for the development of T2DM^15–17^.

Most previous studies assessed baseline WC or short-term changes in WC that fail to fully reflect long-term dynamic patterns and their clinical outcomes. Also, the association between the trend of WC over time and the risk of developing T2DM remains a subject of debate, with limited studies examining WC as a time-dependent or dynamic risk factor. By drawing on data from the Tehran Lipid and Glucose Study (TLGS), a robust longitudinal population-based cohort, we aim to shed light on how changes in WC over time may contribute to T2DM development during an 18 years period by using latent class trajectory analysis.

## Methods

### Study design

The data used in the study were derived from TLGS, a large prospective cohort study initiated in 1999 to explore the prevalence and risk factors for non-communicable diseases. In the TLGS, a sample of 15,005 participants, aged 3 to 69 years, was selected using a multistage stratified cluster random sampling method. The participants were drawn from residents served by three medical health centers in Tehran’s district No. 13. These centers maintained field data for over 90% of the families within their coverage area, ensuring that the sample represented the broader population of Tehran. The follow-up examination visits occurred roughly every three years over an 18 years period. In the present study, we divided the follow-up period into two intervals: a 9 years period to track WC trajectories (measurement period: from the first examination visit to the end of the fourth follow-up visit) and a 9 years period to record T2DM incidence (outcome assessment period: encompassing follow-up examination visits 4thto 6th).

**Fig. 1:**
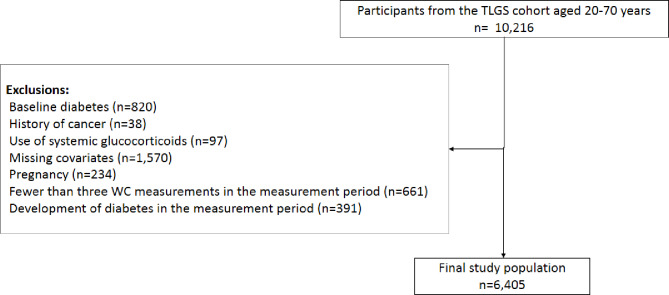
Flowchart of the study population selection. WC, waist circumference; TLGS, Tehran lipid and glucose study.

Among 10,216 individuals aged 20 to 70 years who were enrolled in the study, individuals with baseline diabetes (*n* = 820), history of cancer (*n* = 38), use of systemic glucocorticoids (*n* = 97), missing covariates (*n* = 1570), pregnancy (*n* = 234), fewer than three WC measurements in the measurement period (*n* = 661), development of diabetes in the measurement period (*n* = 391) were excluded. After applying all exclusion criteria, the final study population comprised 6405 normoglycemic participants (Fig. 1).

### Data collection

Data on demographics, medical history, and physical activity were gathered using standardized questionnaires administered by trained staff. Weight and height were recorded using calibrated instruments. WC was evaluated at the level of umbilical in centimeters (cm) while participants were standing in a natural position in light clothing using standardized meters. Fasting plasma glucose (FPG), lipids, and postprandial glucose were measured from blood samples using standardized enzymatic methods, with rigorous quality control procedures to ensure accuracy. Glucose levels were measured using the enzymatic colorimetric method with glucose oxidase. Detailed descriptions of laboratory methods and calibration protocols are provided in a previous publication on the TLGS population^18^.

### Definitions

Incident T2DM was ascertained at each follow-up examination and defined as 2-hour plasma glucose (2-hPG) ≥ 200 mg/dl, FPG ≥ 126 mg/dl, or use of anti-diabetic medication. Prediabetes was defined as a 2-hPG 140–199 mg/dl, FPG 100–125 mg/d. Educational attainment was categorized into higher education (more than 12 years), high school (6 to 12 years), and primary (less than 6 years). Physical activity levels were assessed in Metabolic Equivalent of Task Scale (METS), with low physical activity levels defined as less than 600 min per week. Smoking status was defined as previous or current smoker, including individuals who smoked either daily or occasionally. BMI was computed using the standard equation: weight (kilograms)/height (meters)^2^, and was categorized based on the World Health Organization (WHO) guidelines as follows: obesity (BMI more than 30 kg/m^2^), overweight (BMI: 25–30 kg/m^2^), and normal weight (BMI: 18.5–25 kg/m^2^).

### Statistical analysis

#### Latent trajectory classes of WC

WC trajectories were modeled using latent class trajectory analysis implemented in the lcmm package in R (version 3.3). This approach classifies individuals into distinct latent groups based on their longitudinal patterns. Repeated WC measurements within each class were analyzed using a linear mixed-effects framework.

Trajectory models with 2–5 latent classes were fitted and compared using the Bayesian Information Criterion (BIC) and Akaike Information Criterion (AIC), with lower values indicating better model fit (Supplemental Table S1). A minimum class size of ≥ 5% was also applied to ensure stability and interpretability of the identified trajectories. Although the 5-class model yielded the lowest BIC and AIC values, it generated small and less interpretable classes that did not meet predefined criteria for stability and interpretability. Therefore, the 3-class model was selected as the final solution, based on an optimal balance between model fit, adequate class sizes, parsimony, and clinical interpretability compared with alternative specifications. In addition, both linear and quadratic terms were evaluated to capture potential non-linear patterns over time.

In the TLGS cohort, participants included in and excluded from the analysis had broadly comparable baseline characteristics, as shown in Supplemental Table S2. No meaningful differences were observed across key demographic, anthropometric, and clinical variables, and no visually appreciable differences were detected between the two groups. Therefore, a complete-case analysis was considered appropriate, suggesting that selection bias due to exclusion of participants may not influence the study findings. Trajectory classes were identified both overall and within subgroups defined by sex (P for interaction = 0.043), glycemic status (P for interaction = 0.004) and BMI (P for interaction = 0.205). Three distinct WC trajectory patterns were observed: low-increasing (class 1), moderate-increasing (class 2), and high-increasing (class 3).

#### Association between WC trajectories and T2DM

All analyses were stratified by sex. Baseline characteristics were summarized using means (SDs) for continuous variables and counts (percentages) for categorical variables. The associations between WC trajectory classes and incident T2DM were assessed using Cox proportional hazards models, with trajectory group membership as the main independent variable. Hazard ratios (HRs) and 95% confidence intervals (CIs) were estimated. The proportional hazards assumption was evaluated using graphical methods (log–log survival plots) and statistical tests based on Schoenfeld residuals.

Model 1 was adjusted for age and sex. Model 2 further adjusted for educational level, smoking status, physical activity, and family history of DM. Model 3 additionally controlled for baseline BMI, FPG, systolic blood pressure (SBP), total cholesterol (TC), the TG/high-density lipoprotein cholesterol (HDL-C) ratio, and use of antihypertensive or lipid-lowering medication. Model 4 additionally adjusted for time-averaged values of the key time-varying covariates to account for longitudinal changes during follow-up. Continuous variables were entered as mean values over follow-up measurements, while categorical variables were coded using a scoring system to reflect their longitudinal status.

All statistical analyses were conducted using Stata version 14 (StataCorp) and R version 3.0.3 (R Foundation for Statistical Computing). A two-sided P value < 0.05 was considered statistically significant.

### Ethics approval declaration

Ethical approval was obtained for this study from the ethics committee of the Research Institute for Endocrine Sciences, Shahid Beheshti University of Medical Sciences (IRB approval No. IR.SBMU.ENDOCRINE.REC.1404.02) and was conducted in accordance with the Declaration of Helsinki.

Clinical trial number: Not applicable.

Human ethics and consent to participate declarations: Written informed consent to participate was obtained from all participants.

## Results

**Fig. 2:**
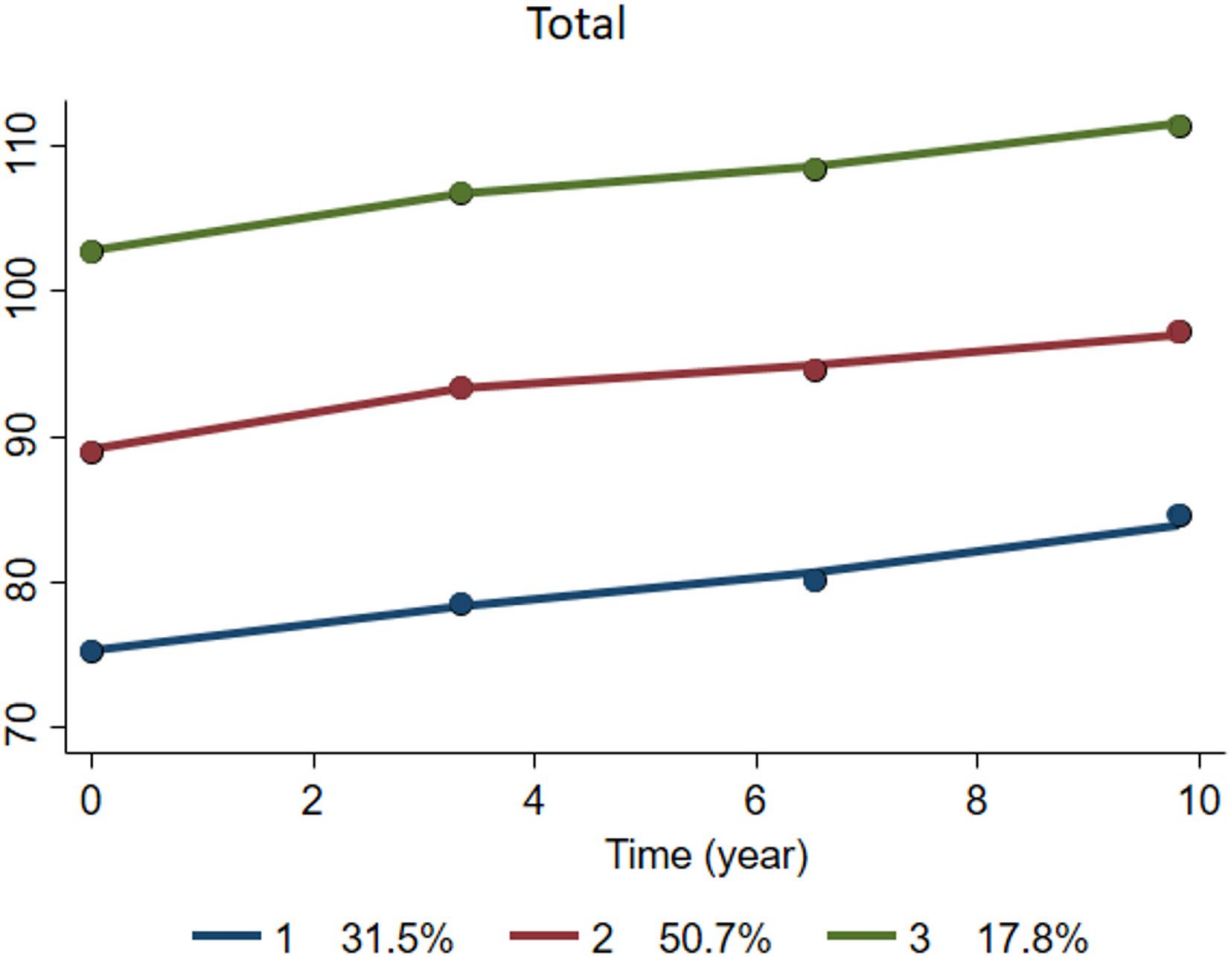
Shows waist circumference trajectory patterns detected over the measurement period. The participants were classified into low-increasing (class 1, blue line), moderate-increasing (class 2, red line), and high-increasing (class 3, green line) groups. The horizontal axis shows time (year), and the vertical axis shows waist circumference (cm).

Among the 6405 normoglycemic individuals enrolled in the study, 2887 (45.1%) were men and 3518 (54.9%) were women. The participants’ average age was 39.7 ± 13.0 years, with a mean WC of 87.0 ± 11.7 (Table 1). Over the follow-up period, 778 new cases of T2DM were documented (Table 2), including 417 (7.9%) cases among individuals with normal glucose levels and 361 (34.0%) cases among those with prediabetes (Table 3). The mean ± SD values of low, moderate, and high-increasing of WC trajectories were 75.0 ± 6.7, 88.9 ± 6.4, 102.9 ± 7.0, respectively (Fig. 2; Table 1).

**Table 1:** Baseline characteristics of the study population according to waist circumference trajectory classes *DM* diabetes, *SBP* systolic blood pressure, *DBP* diastolic blood pressure, *FPG* fasting plasma glucose, *HDL*-*C* high-density lipoprotein cholesterol. *The population was classified into three trajectory classes using latent growth mixture modeling, including trajectories of low-increasing (class 1), moderate-increasing (class 2), and high-increasing (class 3) waist circumference. The categorical and continuous variables were reported as count (percentage) and mean ± SD, respectively.

	Total	WC trajectory classes*			*P* value
		Class 1	Class 2	Class 3	
Number of participants	6405	2019 (31.5)	3248 (50.7)	1138 (17.8)	–
Age (years)	39.74 ± 13.04	34.13 ± 12.01	41.40 ± 12.59	44.97 ± 12.55	< 0.001
Male	2887 (45.07)	619 (30.66)	1670 (51.42)	598 (52.55)	< 0.001
Body mass index (kg/m^2^)	26.50 ± 4.51	22.61 ± 2.96	26.98 ± 3.01	32.04 ± 3.86	< 0.001
Waist circumference (cm)	87.05 ± 11.68	75.02 ± 6.68	88.95 ± 6.45	102.98 ± 7.02	< 0.001
Education					< 0.001
Illiterate/primary school (< 6 years.)	2238 (37.19)	449 (23.37)	1217 (40.15)	572 (53.66)	
High school (6–12 years)	2830 (47.03)	1117 (58.15)	1325 (43.71)	388 (36.40)	
Higher education (≥ 12 years)	950 (15.79)	355 (18.48)	489 (16.13)	106 (9.94)	
Smokers					< 0.001
Never	5071 (79.17)	1697 (84.05)	2491 (76.69)	883 (77.59)	
Former	464 (7.24)	89 (4.41)	269 (8.28)	106 (9.31)	
Current	870 (13.58)	233 (11.54)	488 (15.02)	149 (13.09)	
Low physical activity	4035 (63.00)	1226 (60.72)	2069 (63.70)	740 (65.03)	0.028
DM family history	482 (9.43)	169 (8.65)	243 (10.03)	70 (9.56)	0.23
SBP (mmHg)	115.90 ± 16.64	109.19 ± 13.88	117.59 ± 16.37	122.95 ± 17.72	< 0.001
DBP (mmHg)	76.49 ± 10.33	72.18 ± 9.02	77.47 ± 10.09	81.31 ± 10.32	< 0.001
FPG (mg/dL)	88.80 ± 8.89	86.14 ± 8.32	89.48 ± 8.74	91.60 ± 9.09	< 0.001
Triglyceride (mg/dL)	157.80 ± 99.52	113.35 ± 69.32	171.60 ± 101.22	197.29 ± 111.27	< 0.001
HDL-C (mg/dL)	42.00 ± 10.74	45.75 ± 10.99	40.46 ± 10.11	39.74 ± 10.31	< 0.001
Total cholesterol (mg/dL)	202.41 ± 43.86	186.84 ± 41.35	207.35 ± 42.65	215.91 ± 43.84	<0.001
Anti-hypertensive drug use	241 (3.77)	32 (1.59)	135 (4.16)	74 (6.51)	< 0.001
Lipid-lowering drug use	95 (1.48)	13 (0.64)	51 (1.57)	31 (2.72)	< 0.001

**Table 2:** Sex-stratified Cox proportional hazard ratios (HRs) of the waist circumference trajectory patterns for incidence of type 2 diabetes mellitus.

	Events	IR (95% CI)^*^	HR (95% CI)				
			Unadjusted	Model 1	Model 2	Model 3	Model 4
Men (*n* = 2887)							
Class 1 (*n* = 619)	22	2.6 (1.7–3.9)	1.0 (Reference)	1.0 (Reference)	1.0 (Reference)	1.0 (Reference)	1.0 (Reference)
Class 2 (*n* = 1670)	210	9.1 (8.0–10.4)	3.64 (2.35–5.66)	3.41 (2.20–5.29)	3.46 (2.23–5.36)	2.45 (1.53–3.95)	1.63 (1.02–2.62)
Class 3 (*n* = 598)	120	15.2 (12.7–18.1)	6.52 (4.14–10.27)	5.93 (3.76–9.34)	5.90 (3.74–9.30)	3.04 (1.68–5.48)	2.04 (1.12–3.73)
Women (*n* = 3518)							
Class 1 (*n* = 1400)	69	3.4 (2.7–4.4)	1.0 (Reference)	1.0 (Reference)	1.0 (Reference)	1.0 (Reference)	1.0 (Reference)
Class 2 (*n* = 1578)	204	9.2 (8.0–10.5)	2.79 (2.12–3.66)	2.40 (1.80–3.19)	2.43 (1.83–3.23)	1.50 (1.09–2.08)	1.97 (1.02–2.62)
Class 3 (*n* = 540)	153	21.4 (18.3–25.1)	7.30 (5.49–9.70)	5.71 (4.18–7.81)	5.75 (4.21–7.86)	2.36 (1.52–3.67)	2.48 (1.78–3.45)
Total (*n* = 6405)							
Class 1 (*n* = 20,19)	91	3.2 (2.6–3.9)	1.0 (Reference)	1.0 (Reference)	1.0 (Reference)	1.0 (Reference)	1.0 (Reference)
Class 2 (*n* = 3248)	414	9.1 (8.3–10.1)	3.01 (2.40–3.78)	2.61 (2.07–3.29)	2.60 (2.07–3.28)	1.69 (1.30–2.19)	1.69 (1.33–2.15)
Class 3 (*n* = 1138)	273	18.1 (16.1–20.4)	6.54 (5.16–8.30)	5.25 (4.10–6.71)	5.21 (4.07–6.67)	2.35 (1.67–3.30)	2.26 (1.75–2.93)

Model 1: Adjusted for age and. Model 2: Adjusted for age, education, smoking, physical activity, DM family history. Model 3: Adjusted for age, education, smoking, DM family history, physical activity, baseline BMI, anti-hypertensive drug use, lipid-lowering drug use, fasting plasma glucose, systolic blood pressure, total cholesterol, and triglyceride/ high density lipoprotein-cholesterol. Model 4: adjusted for time-averaged or time-updated covariates during follow-up. Continuous variables were included as mean values over follow-up (age, BMI, fasting plasma glucose, systolic blood pressure, total cholesterol, triglyceride/ high density lipoprotein-cholesterol), while categorical variables were incorporated using scoring approaches based on their longitudinal status (smoking status, anti-hypertensive drug use, and lipid-lowering drug use).

*The population was classified into three trajectory classes using latent growth mixture modeling, including trajectories of low-increasing (class 1), moderate-increasing (class 2), and high-increasing (class 3) waist circumference. HR hazard ratios, **IR Incidence rate per 1000 person-years, CI confidence interval.

**Table 3:** Cox proportional hazard ratios (HRs) of the waist circumference trajectory patterns for incidence of type 2 diabetes mellitus in normoglycemic and prediabetic individuals.

	Events	IR (95% CI)*	HR (95% CI)			
			Unadjusted	Model 1	Model 2	Model 3
Normoglycemic subjects at baseline (*n* = 5245)						
Class 1 (*n* = 1755)	50	2.0 (1.5–2.7)	1.0 (Reference)	1.0 (Reference)	1.0 (Reference)	1.0 (Reference)
Class 2 (*n* = 2599)	221	6.0 (5.3–6.9)	3.11 (2.29–4.22)	2.86 (2.09–3.92)	2.86 (2.09–3.91)	1.82 (1.28–2.59)
Class 3 (*n* = 891)	146	12.1 (10.3–14.3)	6.80 (4.93–9.38)	5.95 (4.26–8.33)	5.94 (4.24–8.31)	2.58 (1.63–4.09)
Prediabetic subjects at baseline (*n* = 1160)						
Class 1 (*n* = 311)	62	14.5 (11.3–18.6)	1.0 (Reference)	1.0 (Reference)	1.0 (Reference)	1.0 (Reference)
Class 2 (*n* = 642)	211	24.8 (21.7–28.4)	1.92 (1.45–2.56)	1.83 (1.37–2.44)	1.85 (1.39–2.46)	1.44 (1.03–2.00)
Class 3 (*n* = 207)	88	34.7 (28.2–42.8)	3.00 (2.17–4.16)	2.79 (2.01–3.89)	2.85 (2.04–3.97)	1.66 (1.01–2.74)

Model 1: Adjusted for age and sex. Model 2: Adjusted for age, sex, education, smoking, physical activity, DM family history. Model 3: Adjusted for age, sex, education, smoking, DM family history, physical activity, baseline BMI, anti-hypertensive drug use, lipid-lowering drug use, fasting plasma glucose, systolic blood pressure, total cholesterol, and triglyceride/ high-density lipoprotein cholesterol.

*The population was classified into three trajectory classes using latent growth mixture modeling, including trajectories of low-increasing (class 1), moderate-increasing (class 2), and high-increasing (class 3) waist circumference. *HR* hazard ratios, **IR Incidence rate per 1000 person-years, *CI* confidence interval.

Baseline characteristics were summarized in Table 1 according to the WC trajectory class of the study participants. Participants in the high-increasing WC trajectory class had higher mean baseline values for BMI, WC, BP, FPG, TG, and TC than those in the low-increasing WC trajectory class. Moreover, the high-increasing WC trajectory class included a greater proportion of men, and individuals with lower education.

Table 2 presented sex-specific HR (95% CI) for the association of incidence T2DM with WC trajectory classes’ patterns. In the total population, HRs (95%CI) for T2DM were 1.7 (1.3–2.1) and 2.3 (1.7–2.9), respectively for the moderate- and high-increasing WC trajectory class in the fully-adjusted model. In fully adjusted model, HR (95% CI) of T2DM in men and women within the high increasing WC trajectory class were 2.0 (1.1–3.7) and 2.3 (1.8–3.4), respectively.

**Fig. 3:**
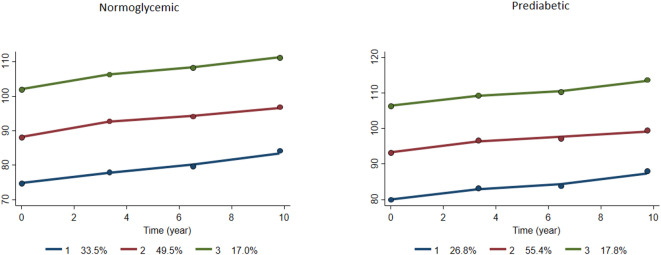
Shows waist circumference trajectory patterns detected over the measurement period in the normoglycemic and prediabetes subgroups, separately. The horizontal axis shows time (year), and the vertical axis shows waist circumference (cm)..

WC trajectory patterns were also identified within the normoglycemic and prediabetic subgroup (Fig. 3; Table 3). For normoglycemic participants in the fully-adjusted model, the high-increasing WC trajectory class had an HR (95% CI) of 2.6 (1.6–4.1), while prediabetic participants in the same WC trajectory class had an HR (95% CI) of 1.7 (1.0–2.7) in the fully-adjusted model.

**Fig. 4:**
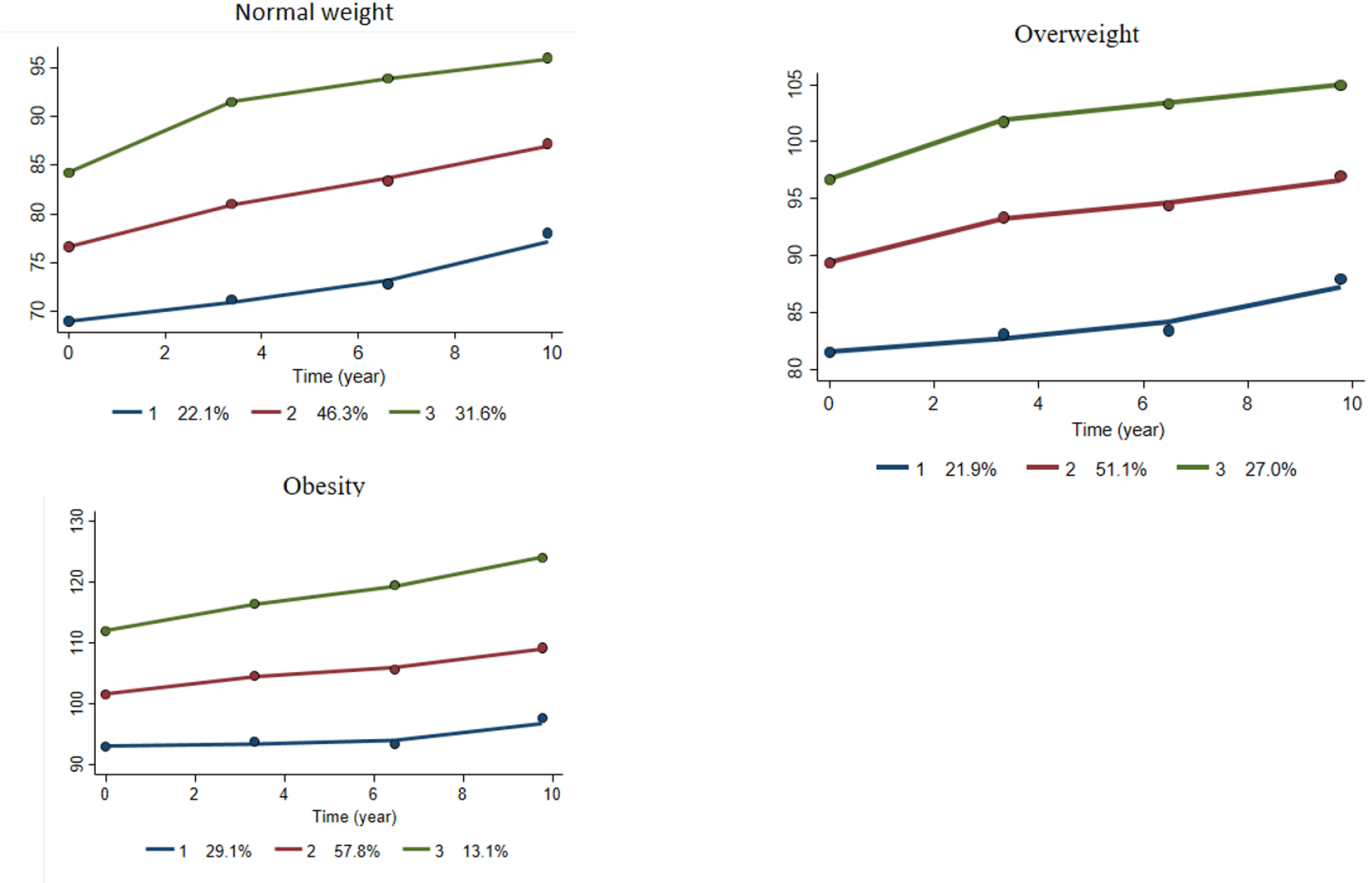
Shows waist circumference trajectory classes for baseline body mass index group (normal weight, overweight, obesity) separately. The horizontal axis shows time (year), and the vertical axis shows waist circumference (cm).

Table 4 presented the HR (95% CI) of the identified WC trajectory patterns for incidence of T2DM based on baseline weight status. In this analysis, WC trajectory class 1 within each BMI group was considered as the reference category. In the normal weight category, the HR (95% CI) of T2DM in WC trajectory class 2 was 1.6 (0.8–3.1), and for WC trajectory class 3, it was 2.5 (1.2–5.4) for developing T2DM in fully-adjusted model. In the overweight group, the HR (95% CI) for WC trajectory class 2 was 1.4 (0.9–2.0), and for WC trajectory class 3 it was 2.1 (1.4–3.2) for developing T2DM in fully-adjusted model. In the obese group, the HR (95% CI) for WC class 2 was 1.8 (1.3–2.6) and for WC class 3 it was 3.3 (2.0–5.4) in fully-adjusted model (Fig. 4).

**Table 4:** Cox proportional hazard ratios (HRs) of the waist circumference trajectory patterns for incidence type 2 diabetes mellitus in baseline weight status.

	Events	IR (95% CI)*	HR (95% CI)			
			Unadjusted	Model 1	Model 2	Model 3
Normal weight (*n* = 2471)						
Class 1 (*n* = 545)	13	1.7 (1.0–2.9)	1.0 (Reference)	1.0 (Reference)	1.0 (Reference)	1.0 (Reference)
Class 2 (*n* = 1,147)	60	3.7 (2.9–4.8)	2.29 (1.26–4.17)	1.95 (1.06–3.58)	1.89 (1.02–3.47)	1.59 (0.82–3.08)
Class 3 (*n* = 779)	78	7.2 (5.8–9.0)	4.66 (2.59–8.39)	3.50 (1.87–6.53)	3.43 (1.83–6.40)	2.55 (1.20–5.40)
Overweight (*n* = 2636)						
Class 1 (*n* = 577)	40	4.9 (3.6–6.7)	1.0 (Reference)	1.0 (Reference)	1.0 (Reference)	1.0 (Reference)
Class 2 (*n* = 1,349)	168	8.9 (7.6–10.3)	1.89 (1.34–2.67)	1.55 (1.08–2.22)	1.53 (1.07–2.20)	1.40 (0.97–2.02)
Class 3 (*n* = 710)	145	15.1 (12.8–17.7)	3.35 (2.36–4.76)	2.60 (1.77–3.81)	2.55 (1.74–3.76)	2.13 (1.40–3.23)
Obesity (*n* = 1298)						
Class 1 (*n* = 378)	49	9.1 (6.9–12.0)	1.0 (Reference)	1.0 (Reference)	1.0 (Reference)	1.0 (Reference)
Class 2 (*n* = 749)	165	16.5 (14.1–19.2)	1.95 (1.42–2.68)	2.03 (1.46–2.82)	2.06 (1.48–2.86)	1.85 (1.31–2.61)
Class 3 (*n* = 171)	60	27.4 (21.3–35.3)	3.68 (2.52–5.37)	3.92 (2.64–5.83)	3.94 (2.65–5.86)	3.29 (2.02–5.36)

Model 1: Adjusted for age and sex. Model 2: Adjusted for age, sex, education, smoking, physical activity, DM family history. Model 3: Adjusted for age, sex, education, smoking, DM family history, physical activity, baseline BMI, anti-hypertensive drug use, lipid-lowering drug use, fasting plasma glucose, systolic blood pressure, high-density lipoprotein cholesterol, total cholesterol, and triglyceride/HDL-C.

*The population was classified into three trajectory classes using latent growth mixture modeling, including trajectories of low-increasing (class 1), moderate-increasing (class 2), and high-increasing (class 3) waist circumference. *HR* hazard ratios, **IR Incidence rate per 1000 person-years, *CI* confidence interval.

Table 5 presented the HR (95% CI) for incident T2DM according to both baseline weight status and WC trajectory patterns. In this analysis, normal weight individuals within class 1 WC trajectory were considered the reference category. The HRs (95% CI) of T2DM for normal weight, overweight, and obesity in class 3 WC trajectory in fully-adjusted model compared with normal weight class 1 WC trajectory as the reference were 3.11 (1.8–5.7), 5.1 (2.9–9.1), and 10.7 (5.8–19.7), respectively. The HRs (95% CI) of T2DM for normal weight, overweight, and obesity within class 2 WC trajectory in fully-adjusted model compared with normal weight class 1 WC trajectory as a reference were 1.8 (0.9–3.3), 3.1 (1.8–5.6), and 5.1 (2.9–9.2), respectively.

**Table 5:** Cox Proportional Hazard Ratios (HRs) for incident type 2 diabetes mellitus according to baseline weight status and waist circumference trajectory patterns.

	HR (95% CI)			
	Unadjusted	Model 1	Model 2	Model 3
Normal weight Class 1 (*n* = 545)	1.0 (Reference)	1.0 (Reference)	1.0 (Reference)	1.0 (Reference)
Normal weight Class 2 (*n* = 1,147)	2.28 (1.25–4.15)	2.03 (1.11–3.71)	2.04 (1.12–3.72)	1.81 (0.99–3.31)
Normal weight Class 3 (*n* = 779)	4.64 (2.58–8.36)	3.79 (2.08–6.88)	3.78 (2.08–6.87)	3.11 (1.71–5.66)
Overweight Class 1 (*n* = 577)	3.00 (1.60–5.60)	2.67 (1.43–5.00)	2.69 (1.44–5.03)	2.04 (1.09–3.82)
Overweight Class 2 (*n* = 1,349)	5.68 (3.23–9.98)	4.44 (2.51–7.85)	4.45 (2.52–7.86)	3.14 (1.77–5.57)
Overweight Class 3 (*n* = 710)	10.06 (5.71–17.75)	7.66 (4.29–13.65)	7.62 (4.27–13.60)	5.12 (2.86–9.16)
Obesity Class 1 (*n* = 378)	5.78 (3.14–10.66)	4.59 (2.48–8.48)	4.59 (2.48–8.49)	3.00 (1.62–5.58)
Obesity Class 2 (*n* = 749)	11.31 (6.43–19.89)	8.42 (4.76–14.90)	8.40 (4.75–14.85)	5.15 (2.89–9.17)
Obesity Class 3 (*n* = 171)	21.23 (11.65–38.69)	15.61 (8.51–28.63)	15.52 (8.46–28.48)	10.72 (5.81–19.75)

Model 1: Adjusted for age and sex. Model 2: Adjusted for age, sex, education, smoking, physical activity, DM family history. Model 3: Adjusted for age, sex, education, smoking, DM family history, physical activity, anti-hypertensive drug use, lipid-lowering drug use, fasting plasma glucose, systolic blood pressure, high-density lipoprotein cholesterol, total cholesterol, and triglyceride/HDL-C.

*The population was classified into three trajectory classes using latent growth mixture modeling, including trajectories of low-increasing (class 1), moderate-increasing (class 2), and high-increasing (class 3) waist circumference. *HR* hazard ratios, **IR Incidence rate per 1000 person-years, *CI* confidence interval.

## Discussion

In this large prospective cohort study, using latent class trajectory analysis, we determined three distinct WC trajectory patterns (low-increasing, moderate-increasing, and high-increasing) and demonstrated a strong association between high-increasing WC trajectory class and the risk of developing T2DM over an 18-year follow-up period in an adult Iranian population. This pattern was consistent across sex, glycemic status, and baseline weight categories. These findings suggest that longitudinal changes in central adiposity, rather than static anthropometric measures alone, may provide important information regarding T2DM risk.

Compared with BMI, which measures overall obesity, WC is a clinical marker that more accurately reflects the accumulation of visceral fat in the abdominal area, and is highly relevant to the pathophysiology of T2DM^19,20^. Visceral adipose tissue is metabolically active and secretes various pro-inflammatory cytokines, as well as adipokines and free fatty acids. These substances create a state of chronic low-grade inflammation and increase insulin resistance by impairing insulin signaling pathways. Excess free fatty acids released from visceral fat also increase hepatic glucose production and disrupt the regulation of glucose homeostasis, further promoting hyperglycemia^21,22^.

Previous studies have assessed the association between WC or its changes with the risk of T2DM^16,23–25^; while the association between WC trajectories and the development of T2DM has been less investigated^26,27^. A study of 35,643 Danish individuals in 2011 (Danish Diet Cancer and Health Study) showed that baseline WC rather than WC changes during 5.4 years was associated with the incidence of T2DM^16^.In the current study a high-increasing WC trajectory class was associated with a two-fold increase in the risk of developing T2DM (Table 2). In line with our findings, Jeon et al.'s study (2019) on 4992 individuals aged over 40 years in Korea identified five WC trajectory groups using the group-based trajectory model (GBTM) and reported that participants with sharply increasing WC trajectories had a 5-7-fold higher risk of developing T2DM during 14 years of follow-up^27^ .

To our knowledge, this study is among the first to assess the link between trajectories of WC and the incidence of T2DM separately in normoglycemic and prediabetic individuals. In the fully-adjusted model, a high-increasing WC trajectory class was associated with about a 2.5-fold higher risk of developing T2DM among normoglycemic individuals, compared with roughly a 60% increased risk in the prediabetic group (Table 3). The lower risk observed in the prediabetic group relative to the normoglycemic group may reflect that individuals with prediabetes already carry a higher baseline risk of progression to T2DM; thus, WC increases may have a relatively smaller incremental impact. Conversely, in normoglycemic individuals, an increase in WC may act as a stronger early disruptor of metabolic balance. Additionally, prediabetic individuals may adopt better preventive behaviors. Also, differences in subgroup sizes, residual confounding, or limited precision due to smaller class size may have influenced these results. A study conducted in the TLGS examined the association between BMI trajectories and the incidence of T2DM, which found that a high-increasing BMI trajectory class was associated with about a 4-fold increase in the risk of T2DM in normoglycemic individuals compared to about a 3-fold increased risk of T2DM in prediabetic individuals^28^. In a large Japanese cohort with prediabetes, those who progressed to T2DM showed a faster annual increase in BMI and WC, whereas individuals who returned to normoglycemia had the lowest baseline BMI and WC with minimal change across time^29^. The strongest association in the normoglycemic group highlights the value of early prevention of central obesity even before dysglycemia develops.

Our findings indicated that an increase in WC was significantly associated with a higher risk of developing T2DM in both sexes, and men exhibited a stronger association than women (HR 3.04; 95% CI 1.7–5.5 vs. 2.4; 95% CI 1.5–3.7) (Table 2). Jeon et al. reported that all WC trajectory groups were associated with an increased risk of T2DM in women, but in men, only groups with a high-increasing WC trajectory class had a higher risk of developing T2DM . In a study conducted in women in the TLGS, women in the highest WC trajectory class had a 2.3-fold higher risk compared to those in the lowest WC class^30^. Sex differences may be due to biological factors, differences in fat storage pattern and hormonal status.

In our study, participants with high-increasing WC trajectory class in each BMI category (normal, overweight, obesity) had a higher risk of T2DM than participants with low-increasing WC trajectory class (Table 4). In addition, different WC trajectory classes were examined across various weight groups, while individuals in WC trajectory class 1 with normal weight were regarded as the reference group (Table 5). We found that the risk of developing T2DM was 10-fold higher in obese individuals within WC trajectory class 3 compared to the reference group, which shows that coexistence of central and general obesity heightened the risk of T2DM. Moreover, the risk of T2DM in obese individuals within WC trajectory class 1 was approximately similar to that of normal weight individuals within WC trajectory class 3 and overweight individuals within WC trajectory class 2. Another study in China on 54,434 participants showed that odds ratio (95% CI) for individuals who had moderate/high WC and high BMI trajectory was 3.9 (3.5–4.5) and for individuals who had moderate/high WC and a low BMI trajectory was 3.0 (2.3–3.9) for the risk of developing T2DM^26^. These findings highlight the importance of considering both BMI and WC together for predicting development of T2DM; this is in line with a recent redefinition of obesity in 2025 suggesting that BMI alone is not sufficient to determine an individual’s health status. They suggested that obesity is best defined with at least one of the following indicator added to BMI including a direct measurement of body fat, WC, waist to hip ratio, and waist-to-height ratio^31^.

Several limitations warrant consideration. First, despite the robust longitudinal design and adjustment for numerous confounders, residual confounding by unmeasured variables such as dietary intake, sleep quality, or genetic susceptibility, cannot be ruled out. Second, the study population comprised urban Iranian adults, potentially limiting generalizability to other ethnicities or rural settings with different lifestyle profiles. T2DM events were identified at scheduled follow-up examinations rather than continuously, so the exact date of onset was interval-censored. In addition, some outcome misclassification between visits cannot be excluded. We did not have data for HbA1c and T2DM was diagnosed based on a single laboratory assessment of FPG and 2-hPG. Additionally, while latent class trajectory analysis captures population heterogeneity, the number and shape of trajectory classes are model-dependent and may differ with alternative statistical criteria. The findings have several important implications. This population-based cohort study was carried out on a representative sample of individuals living in Tehran and followed them for 18 years. Clinically, the findings highlight the need for dynamic, rather than static, anthropometric monitoring in routine health assessments.

In conclusion, this study contributes evidence that the increasing trend in WC is strongly associated with the development of T2DM independent of BMI and other important factors for T2DM. These findings suggest that continuous monitoring of central adiposity may help identify individuals with higher T2DM risk.

## Supplementary Information

Below is the link to the electronic supplementary material.

Supplementary Material 1

## Data Availability

The datasets used and/or analyzed during the current study available from the corresponding author on reasonable request.
