# Supplementary material for: Association between trajectories of waist circumference with incidence of type 2 diabetes mellitus, Tehran lipid and glucose study
Source: Sci Rep. 2026 May 13;16:25011. doi: 10.1038/s41598-026-51739-w (PMC13463085; doi:10.1038/s41598-026-51739-w)
Supplement: Supplementary file 1 [file 41598_2026_51739_MOESM1_ESM.docx]

| **Table S1**. Model fit indices for different trajectory class solutions and polynomial specifications | | |
| --- | --- | --- |
| **Number of Classes** | **BIC Value** | **AIC Value** |
| 2 | -82499.75 | -82465.92 |
| 3 | -80145.61 | -80094.88 |
| 4 | -79015.84 | -78948.19 |
| 5 | -78401.00 | -78316.44 |

| **Supplemental Table S2.** Baseline characteristics of the participants included in and excluded from the analysis | | |
| --- | --- | --- |
| **Baseline**  **characteristics** | **Included participants**  **n=6,405** | **Excluded participants**  **n=2,231** |
| Age (years) | 39.74 ± 13.04 | 40.79±15.75 |
| Male | 2887 (45.07) | 917 (46.88) |
| Body mass index (kg/m2) | 26.50 ± 4.51 | 26.11±4.75 |
| Waist circumference (cm) | 87.05 ± 11.68 | 87.39±12.59 |
| Education |  |  |
| Illiterate/primary school (<6 yrs.) | 2238 (37.19) | 582 (35.64) |
| High school (6-12 years) | 2830 (47.03) | 755 (46.23) |
| Higher education (≥12 years) | 950 (15.79) | 296 (18.413) |
| Smokers |  |  |
| Never | 5071 (79.17) | 1361 (76.08) |
| Former | 464 (7.24) | 126 (7.04) |
| Current | 870 (13.58) | 302 (16.88) |
| Low physical activity | 4035 (63.00) | 934 (52.83) |
| DM family history | 482 (9.43) | 148 (11.11) |
| SBP (mmHg) | 115.90 ± 16.64 | 119.07 ± 20.01 |
| DBP (mmHg) | 76.49 ± 10.33 | 75.86 ± 10.47 |
| FPG (mg/dL) | 88.80 ± 8.89 | 89.29 ± 9.26 |
| Triglyceride (mg/dL) | 157.80 ± 99.52 | 158.96 ± 108.48 |
| HDL-C (mg/dL) | 42.00 ± 10.74 | 43.02 ± 11.03 |
| Total cholesterol (mg/dL) | 202.41 ± 43.86 | 197.57 ± 46.14 |
| Anti-hypertensive drug use | 241 (3.77) | 110 (5.80) |
| Lipid-lowering drug use | 95 (1.48) | 49 (2.51) |
| The categorical and continuous variables were reported as count (percentage) and mean ± SD, respectively. Abbreviations: DM, diabetes; SBP, systolic blood pressure; DBP, diastolic blood pressure; FBS, fasting blood sugar, HDL-C, high-density lipoprotein cholesterol | | |
